# An Orthodontic Approach for the Correction of Transposition along with Multiple Impacted Teeth

**DOI:** 10.1155/2023/6252389

**Published:** 2023-04-06

**Authors:** Sanjay Prasad Gupta, Jamal Giri, Basanta Kumar Shrestha

**Affiliations:** ^1^Department of Orthodontics and Dentofacial Orthopedics, Tribhuvan University Teaching Hospital, Maharajgunj Medical Campus, Institute of Medicine, Tribhuvan University, Kathmandu, Nepal; ^2^Department of Orthodontics, B. P. Koirala Institute of Health Sciences, Dharan, Nepal; ^3^Department of Orthodontics and Dentofacial Orthopedics, Chitwan Medical College, Bharatpur, Nepal

## Abstract

Impaction of teeth affects patients' esthetics, speech, and masticatory efficiency. In addition, the transposition of teeth makes a case difficult to manage. This case report describes a case of a 14-year-old boy with the impaction of the maxillary right central incisor and canine along with the transposition of the right canine and lateral incisor. The impacted teeth were surgically exposed and guided into the arch using orthodontic traction. Likewise, the transposition was corrected orthodontically by moving the teeth to their desired position without any detrimental effect on the adjacent teeth. The patient's esthetics and occlusion improved substantially after the orthodontic intervention.

## 1. Introduction

Tooth impaction is an abnormal condition in which a tooth fails to erupt in its functional position beyond its eruption time. In the maxillary arch, the impaction of canine is the second most common impaction next to the third molar [1]. Maxillary incisor is rarely impacted, but the resulting malocclusion is conspicuous, adversely affecting the smile esthetics and phonetics among others, which could result in low self-esteem among patients [2]; thus, requires early orthodontic intervention. Tooth impaction may also lead to several pathologic conditions like external root resorption of the adjacent teeth, dentigerous cyst, and odontogenic tumors [3, 4].

There are various local etiological factors, which obliterate the eruptive path leading to impaction, such as insufficient space, bony or soft tissue resistance, cysts, and tumors [5–7]. Whereas, other general conditions include osteopetrosis, hypothyroidism, Apert syndrome, and cleidocranial dysplasia [8, 9].

Tooth transposition is a positional interchange of two adjacent teeth within the same quadrant of the dental arch. It is identified as complete transposition when the crowns and the roots of the involved teeth exchange places in the dental arch, and incomplete transposition (pseudo-transposition) when the crowns are transposed, but the roots remain in their normal positions [10].

Transposition of tooth is relatively uncommon with an overall prevalence of 0.33% [11]. Likewise, the prevalence rate of transposition among Nepalese orthodontic patients is 0.66% [12]. It is mostly unilateral, involves the maxillary arch and has a strong genetic component [11]. Transposition most commonly involves the maxillary canine [13] with the transposition of canine and first premolar being the most common form of transposition in the maxillary arch followed by the transposition of canine and lateral incisor [14].

Orthodontic movement of transposed teeth complicated by impaction to their normal positions is quite challenging because of root interference and resorption of adjacent teeth and difficulties in controlling root inclination. Hence, in cases of complete transposition, it is typically recommended that the teeth are aligned in their transposed positions. Nevertheless, it is essential to reshape the crowns of the teeth to obtain an aesthetically pleasing outcome [15].

This case report depicts a 14-year-old Nepali male with impacted right maxillary central incisor and canine complicated by a complete transposition of right maxillary canine and lateral incisor. The impacted and transposed teeth were moved orthodontically to their normal positions using precise biomechanics with minimal detrimental effect to the teeth and adjacent structures. Later, the torque control of the lateral incisor was achieved using various mechanics including the use of custom-made warren spring.

## 2. Case Presentation

### 2.1. Diagnosis and Etiology

A 14-year-old male patient was referred for an orthodontic consultation to the Department of Orthodontics and Dentofacial Orthopedics, Tribhuvan University Teaching Hospital, Kathmandu, Nepal. His chief complaint was a gap and missing teeth in the front region of the upper jaw. The boy had no relevant family history, no significant prenatal and medical history, and no history of parafunctional habits. However, there was a history of trauma to the upper front teeth at the age of 4 years. He was very conscious of the gap present between the teeth and the resulting unesthetic smile.

**Figure 1:**
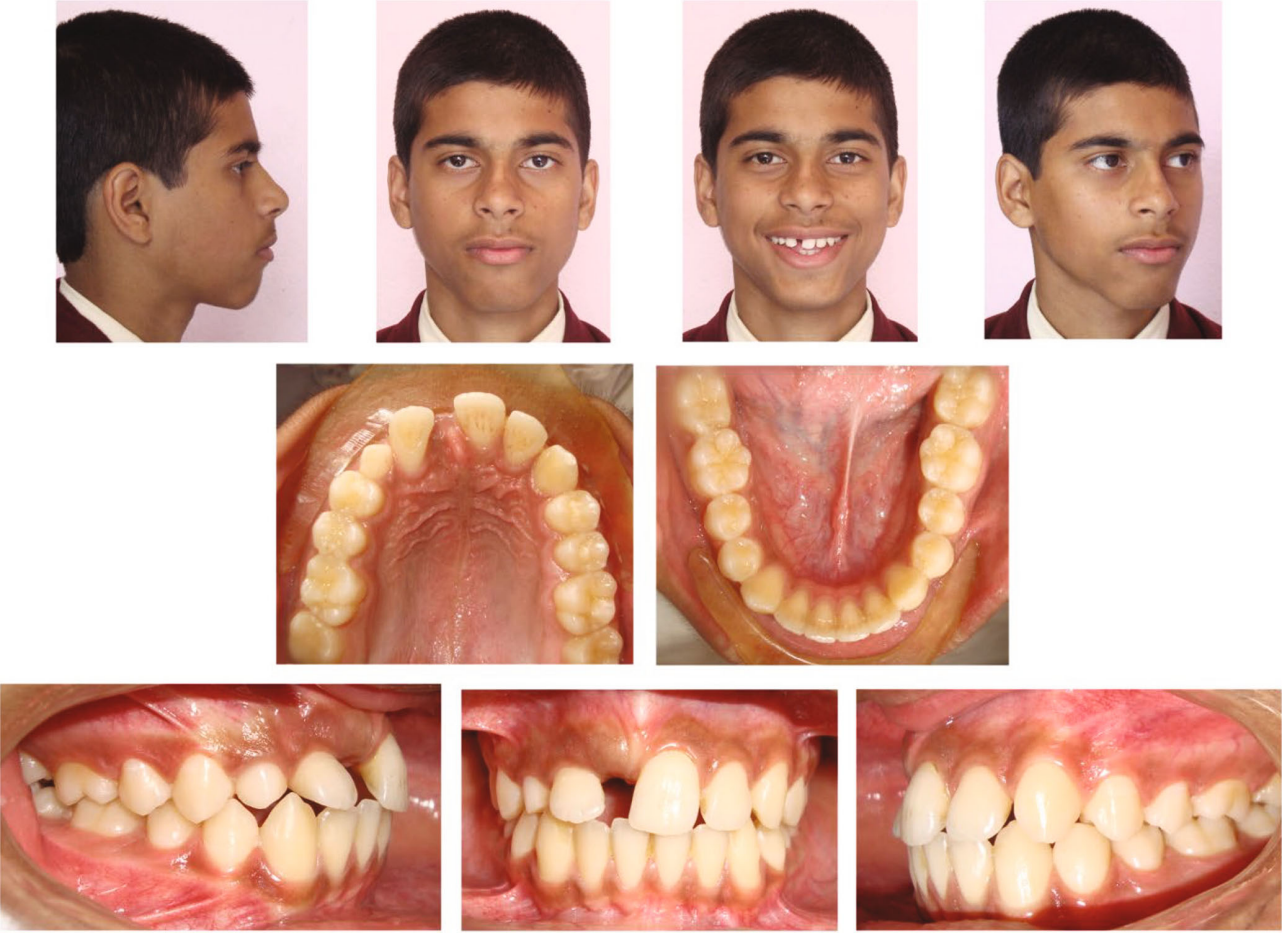
Pretreatment intraoral and extraoral photographs.

On clinical examination, he had a convex profile with apparently symmetric face and competent lips. Intraoral examination revealed Class I molar relationship bilaterally, Class I canine relationship on the left side, and retained right upper deciduous canine with diastema between the teeth in the right upper front region of jaw (Figure 1). He had a reduced overjet (1 mm) and overbite (1.5 mm).

**Figure 2:**
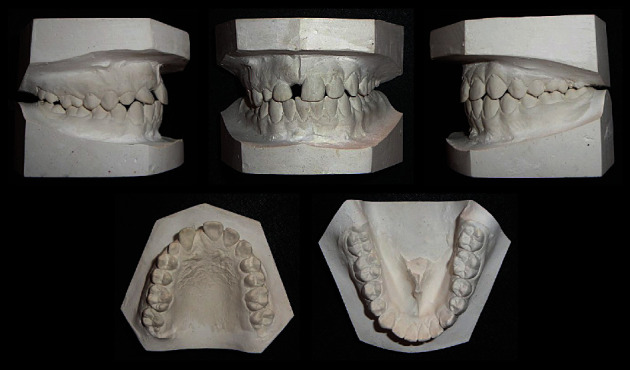
Pre-treatment study models.

Both maxillary and mandibular arches were U-shaped, and spacing was present in the maxillary arch (Figure 2).

**Figure 3:**
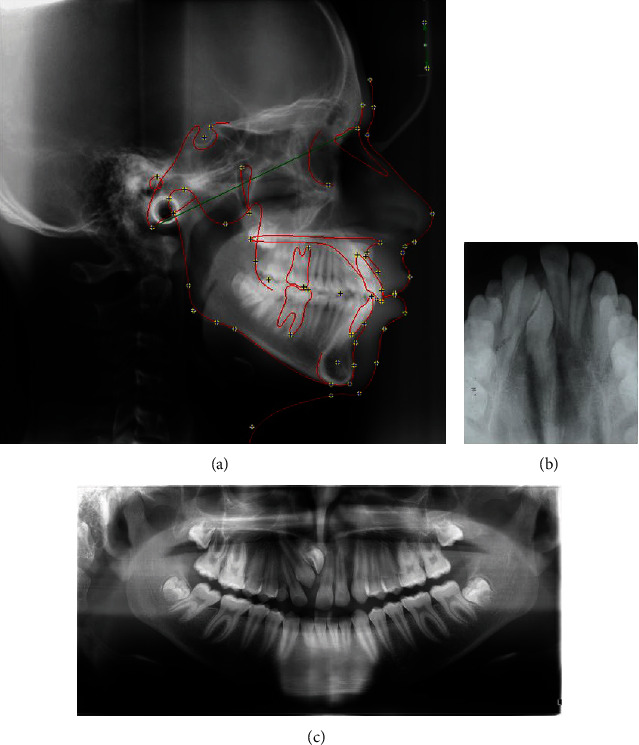
Pre-treatment radiographs. (a) Lateral cephalogram with tracing. (b) Occlusal radiograph. (c) orthopantomogram.

The cephalometric analysis revealed a skeletal Class II relation with an angle formed by the point A, nasion and point B (ANB) of 6° and nearly normal growth pattern (FMA of 26°; SN-GoGn of 29°), proclined and nearly normally placed maxillary incisor and nearly normoclined, and normally placed mandibular incisors with obtuse nasolabial angle (105°) and mildly protrusive lips (Figure 3).

The panoramic and occlusal radiographs confirmed the impaction of the right central incisor and canine along with transposition between the right canine and lateral incisor in the maxillary arch. Third molars were present in all the quadrants, and the overall alveolar bone level was within normal limits (Figure 3).

### 2.2. Treatment Objectives and Alternatives

The following treatment options were offered to the patient:

1. Option 1: Extraction of the right impacted central incisor and canine followed by orthodontic space opening for implant prosthesis. In this option, the patient should wait for at least 4–5 years before implant placement, and there was a great risk of alveolar bone loss. Bone graft and space maintenance might be needed to avoid migration of adjacent and opposing teeth till the implant placement [16].
2. Option 2: Creation of space and surgical exposure of impacted teeth followed by orthodontic traction and alignment of the canine into the transposed lateral incisor position and the right maxillary central incisor into its normal position. Although this treatment option would warrant a shorter treatment time, the maxillary left canine and lateral incisor would need reshaping.
3. Option 3: Creation of space and surgical exposure of impacted teeth followed by orthodontic traction and alignment of the right canine and central incisor into their normal positions. This would preserve the patient's natural teeth and result in better esthetics and occlusion. However, the treatment time would be long, and there was a risk of resorption of adjacent teeth and traction failure.
4. Option 4: Surgical repositioning of the impacted teeth into their positions. Surgical repositioning is usually indicated for impacted teeth in a deep position. However, it is associated with many potential complications, including root resorption, pulp necrosis, and arrest of root formation [17].

After some deliberation, the patient and his parents opted for the third treatment option for better esthetics and occlusion, even though it would require a longer duration of treatment.

The patient had a skeletal Class II relation and already passed the active growth phase; however, his facial profile was not severe enough to undergo orthognathic surgery; hence orthodontic camouflage was planned. A non-extraction treatment approach was planned considering the multiple impacted teeth and acceptable facial profile.

The treatment objectives were as follows:

1. Creation of space for the impacted teeth.
2. Surgical exposure, orthodontic traction, and alignment of impacted and transposed teeth to their desired positions.
3. Achievement of functional occlusion and facial esthetics.

### 2.3. Treatment Progress

**Figure 4:**
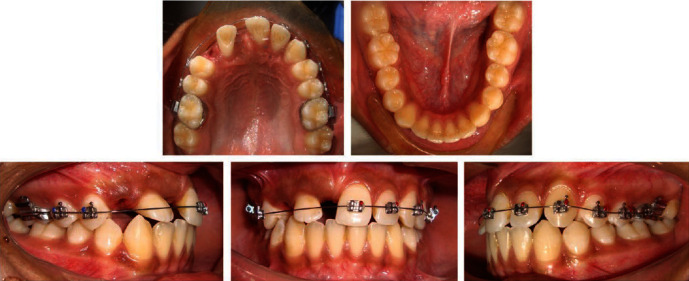
Bonding of brackets and insertion of 0.014″ NiTi archwire in upper arch.

**Figure 5:**
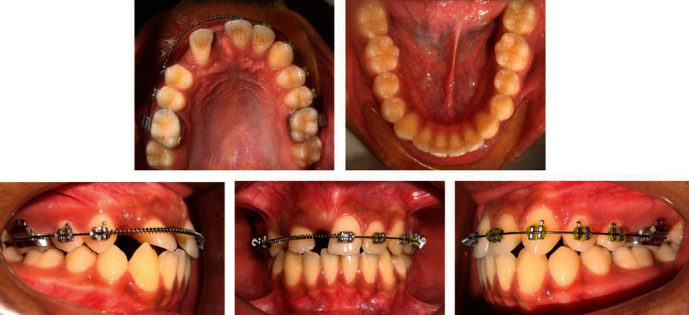
Use of open coil spring for the creation of space.

Extraction of upper right deciduous canine and surgical uncovering of impacted upper right permanent canine and central incisor after levelling and alignment were planned. Initially, maxillary teeth were banded and bonded with fully programmed preadjusted 0.022 slot MBT prescription brackets (Figure 4). The arches were aligned using the following sequence of archwires; 0.014″ NiTi and 0.016″ NiTi. After alignment and levelling, 0.018″ Stainless Steel (SS) Australian archwire (A. J. Wilcock, Whittlesea, VIC, Australia) was placed in the maxillary arch and enough space was opened using an open coil spring (Figure 5). Then the archwire was changed to a rigid 0.019^″^ × 0.025^″^ SS wire with a v-notch in preparation for the surgical uncovering of impacted teeth.

**Figure 6:**
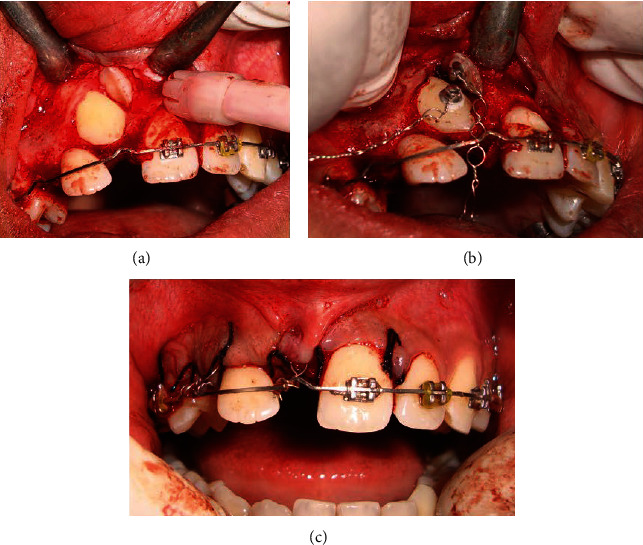
(a) Surgical exposure. (b) Bonding an attachment to impacted canine and central incisor. (c) Flap closure.

A full thickness mucoperiosteal flap was reflected to surgically expose the impacted teeth #11 and #13, and lingual buttons were bonded on the impacted teeth following a strict isolation protocol. Ligature wires with eyelets were tied to the bonded lingual buttons at one end, whereas the other ends were exposed in the desired position of the arch for orthodontic traction before the flap was sutured—a closed eruption technique was used (Figure 6).

**Figure 7:**
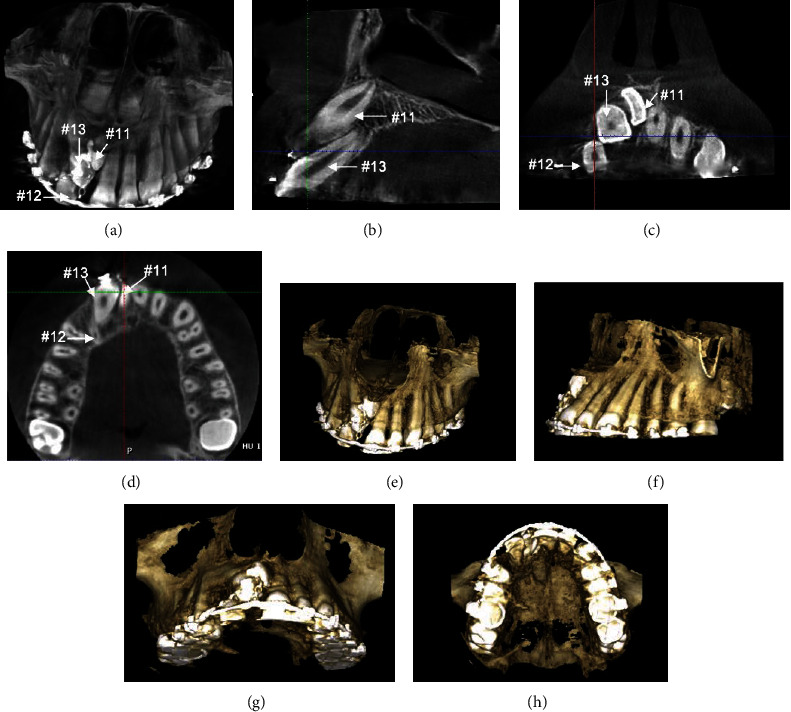
CBCT images of the maxillary anterior region immediately after surgical exposure and attachment bonding: (a) Frontal image. (b) Sagittal image. (c) Coronal image. (d) Axial image. (e–h) Three-dimensional reconstructed images.

Cone-beam computed tomography (CBCT) images taken just after the surgical exposure displayed the precise locations of the impacted teeth along with the condition of the adjacent teeth. There were no signs of resorption of the impacted and the adjacent teeth (Figure 7). CBCT images helped us to track the teeth in desired place by changing the direction of traction force. Precise biomechanical design was used by soldering the hooks in different parts of the archwire based on the desired direction of the force to avoid the root collision during orthodontic traction.

**Figure 8:**
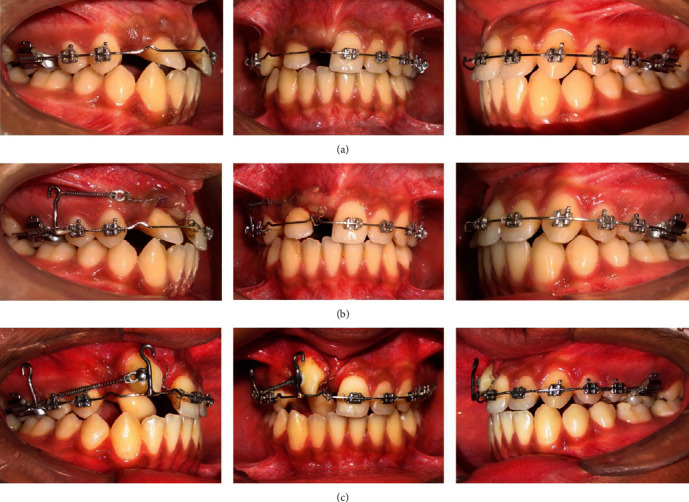
Mid-treatment photographs. (a) Use of 0.019^″^ × 0.025^″^ SS wire with v-notch just before surgical uncovering. (b) Hook soldered on the 0.019″ × 0.025″ SS wire to apply the force in a desired direction after bonding an attachment on the impacted teeth. (c) Retraction of upper right canine distally with the help of closed coil spring.

As teeth #12 and #13 were transposed, tooth #12 was retracted palatally by attaching a bracket on the palatal aspect of tooth #12 to provide enough room labially for the distalization of tooth #13 in its desired position (Figure 8).

**Figure 9:**
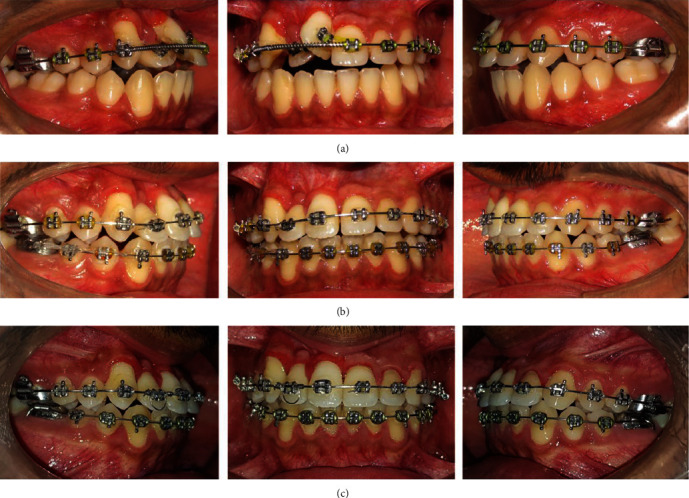
Mid-treatment photographs. (a) Bonding of bracket on the upper right central incisor after its traction. (b) Application of individual labial root torque on 0.019″ x 0.025″ SS archwire in the region of upper right lateral incisor region. (c) Use of custom-made warren spring to achieve additional labial root torque (palatal crown torque) on upper right lateral incisor.

**Figure 10:**
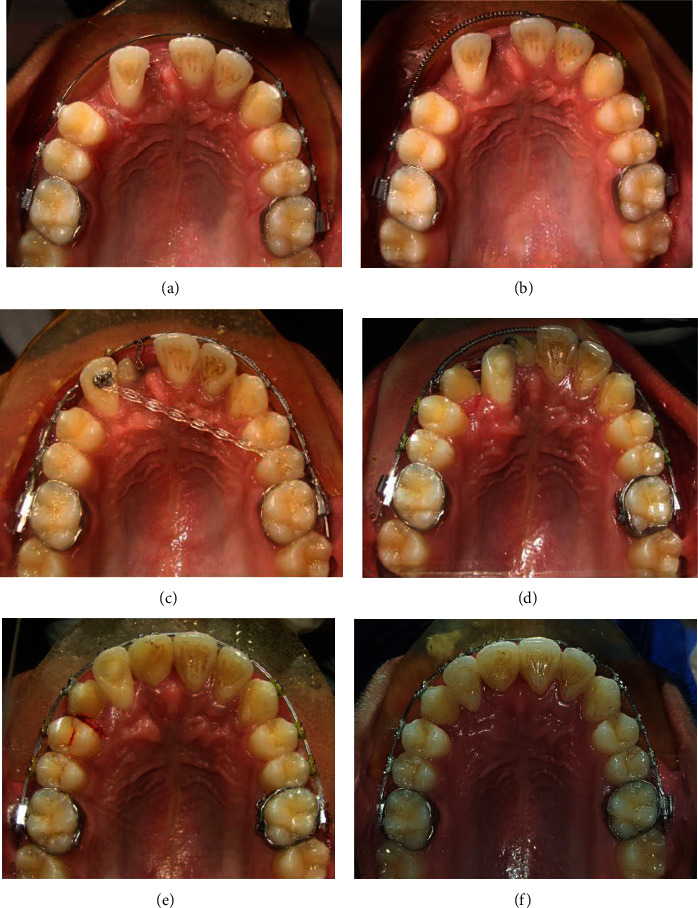
(a–f) Treatment progress as changes seen in the maxillary occlusal photographs.

The labial root torque (palatal crown torque) of tooth #12 was achieved sequentially by inverting the orthodontic bracket upside down, then by adding an individual palatal crown torque in the rectangular wire in the lateral incisor area, and finally by using a custom-made warren spring (a torquing auxiliary made with 0.012″ SS wire) on the rectangular wire (Figures 9 and 10).

### 2.4. Treatment Results

**Figure 11:**
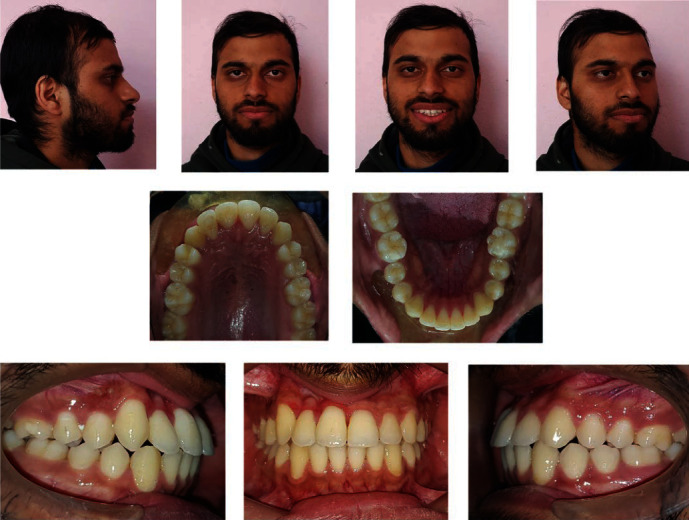
Post-treatment extraoral and intraoral Photographs.

**Figure 12:**
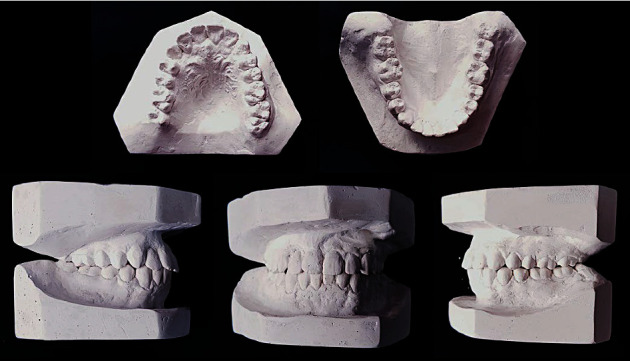
Post-treatment study models.

At the end of the treatment, there was a remarkable improvement in the facial and smile esthetics of the patient with the natural teeth in the desired positions (Figures 11 and 12).

Intraorally, there was nearly normal Angle's Class I molar and canine relationships bilaterally with a normal overjet and overbite. There was canine guidance in lateral excursions with proper anterior guidance without balancing side interferences and the facial midline coincided with both maxillary and mandibular dental midlines.

**Figure 13:**
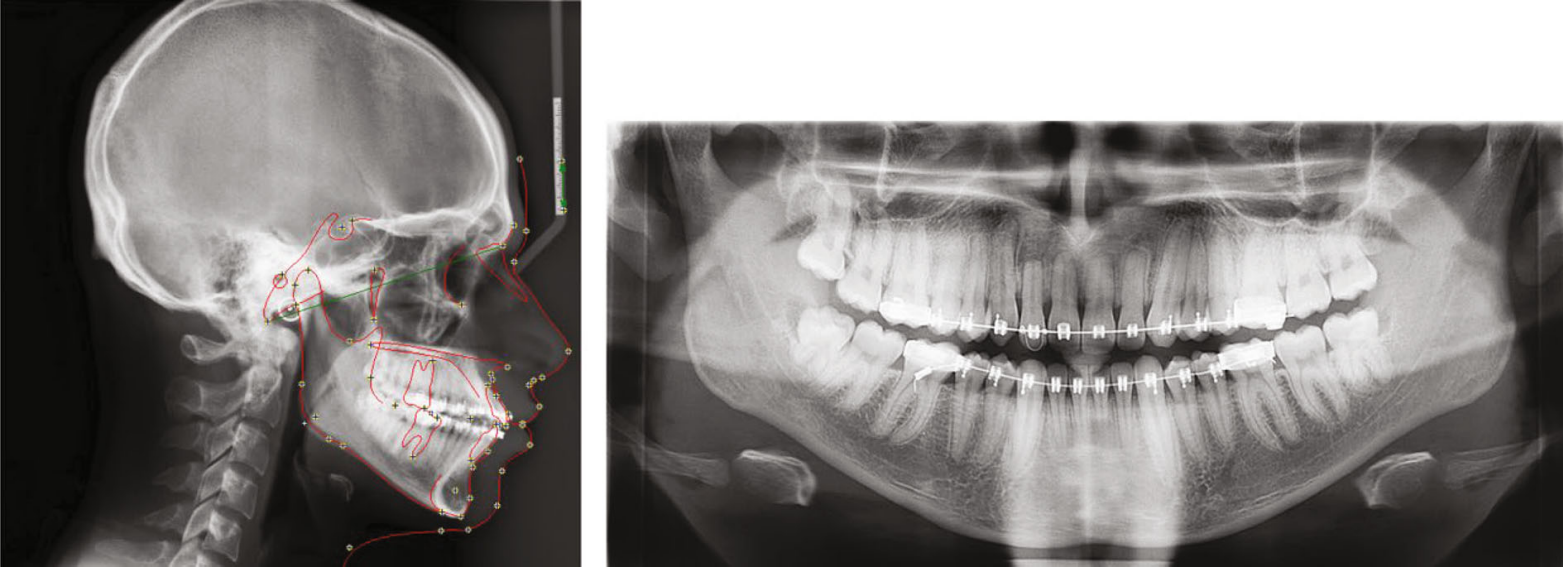
Post-treatment lateral cephalogram with tracing and orthopantomogram.

The post-treatment radiographs (Figure 13) showed changes in the dental and skeletal parameters after the treatment. A panoramic radiograph, which was taken just before debonding to assess the root parallelism showed mild root resorption of tooth #12, but it was clinically acceptable.

**Figure 14:**
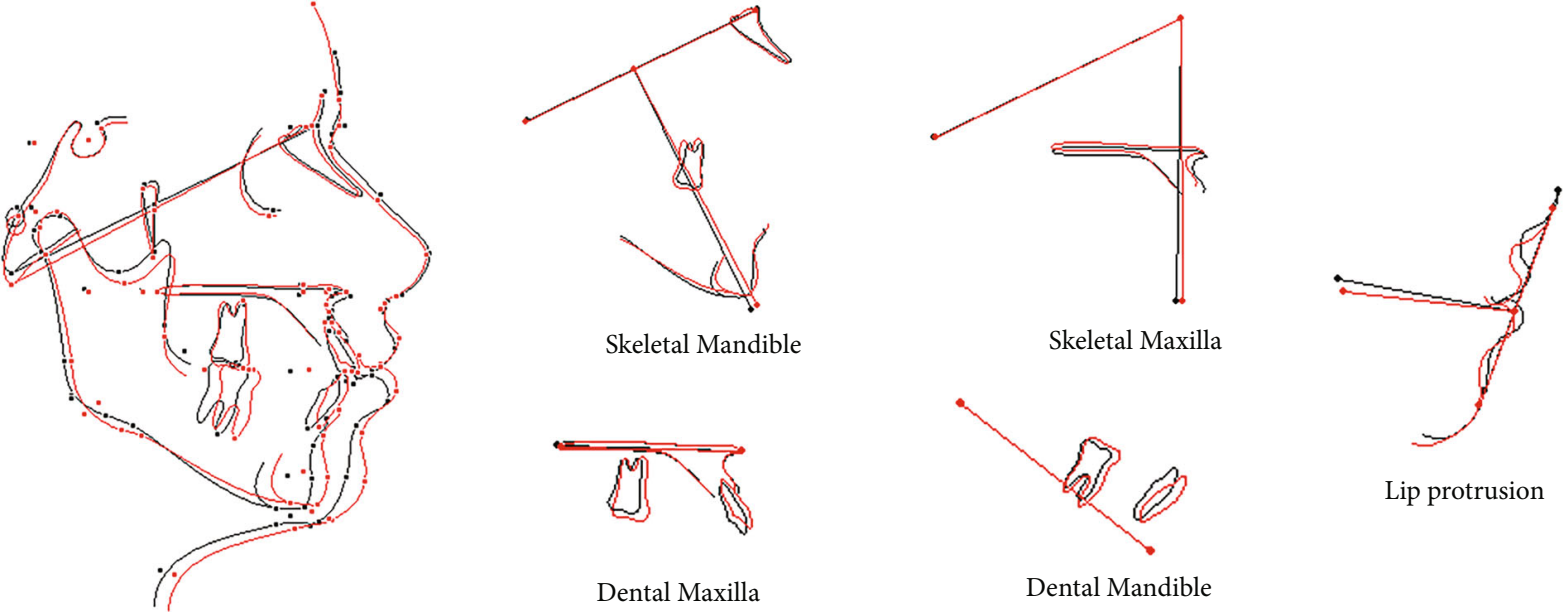
Superimposition of pre-treatment (black line) and post-treatment (red line) cephalometric tracing.

The pre-treatment and post-treatment cephalometric parameters are compared in Table 1, and it is well demonstrated in superimposed tracing (Figure 14).

The patient required a slightly longer course of treatment, around 4 years, but the overall outcome was satisfactory. The patient had a Class I occlusion and improved facial esthetics at the end of the treatment; however, to achieve a symmetric gingival height and to enhance the dento-gingival esthetics, a crown lengthening surgery was advised after the complete cessation of growth.

## 3. Discussion

This case report describes the orthodontic management of multiple impacted maxillary anterior teeth further complicated by transposition using conventional biomechanics. Interference with the adjacent teeth during orthodontic traction was avoided by controlling the force vectors using power arms (soldered hooks) in different parts of the arch wire.

Treatment options for impacted anterior teeth include extraction of the impacted teeth with subsequent prosthetic treatment [18], removal of the eruption resistance and space creation for spontaneous eruption [19], surgical exposure and orthodontic traction [20, 21], and surgical repositioning. Among all, orthodontic traction is considered the most favorable treatment option for impacted teeth as it provides good functional and esthetic results with limited complications [22, 23], but it is relatively demanding technique and time consuming compared with other options [24]. Similarly, tooth transposition is considered as one of the most difficult anomalies to manage and the treatment options for transposition are alignment of teeth in their transposed positions, correction of the teeth to their normal position, and extraction of one or both transposed teeth [25].

In orthodontic literature, very few cases of correction of complete transpositions have been reported [15, 26–28]. The correction is considered complex and could be damaging to both teeth and adjacent structures [10]. Peck and Peck recommended that teeth with pseudo-transposition could be corrected into their normal positions; however, correction was not advocated for the teeth with complete transposition [14].

Factors that need to be considered while managing the transposed case are dental morphology, occlusal considerations, facial esthetics, stage of root development, position of the root apices, patient co-operation, and treatment time [29, 30]. Radiographic evaluation of the patient did not reveal abnormalities with respect to crowns or roots of impacted teeth #11 and #13; therefore, they were deemed suitable for surgical exposure and orthodontic traction into the arch. It was a relatively straightforward decision for the impacted tooth #11, but the same could not be said for tooth #13, which was also transposed with tooth #12. Hence a longer treatment time would be required to align tooth #13 in its normal position in the arch. Treatment time is an important consideration during the management of transposed teeth complicated by impaction [15]. Fortunately, enough cooperation was available from the patient.

A closed eruption technique with light and continuous force was used for the traction of impacted teeth. The closed eruption technique was used because this technique is believed to replicate natural tooth eruption and produce the best esthetic and periodontal outcomes [31]. Elastomeric chains and closed coil springs were used for orthodontic traction, which delivered light and continuous force in the desired direction.

To control the tooth movement in the alveolar bone and to avoid interference between the roots of the teeth, different strategies were used during the treatment. For orthodontic traction of tooth #11, an occlusal force vector was created. Likewise, a disto-occlusal traction was applied via a power arm to the impacted tooth #13 to correct both impaction and transposition. First tooth #12 was retracted palatally by attaching the bracket on the palatal aspect of the right lateral incisor. It provided enough space labially for the distalization of tooth #13 in its desired position. After distalization of tooth #13, proper torque was achieved.

Torque control of an impacted tooth after orthodontic traction and alignment is crucial because the traction force is applied at a single point on the crown; therefore, it is common for the crown to be aligned while the root stays outside the alveolar bone, which requires extra torque control [9, 18].

In this case, proper torque was achieved for all the impacted teeth including tooth #12, which was initially retracted palatally for distalization of impacted tooth #13. The labial root torque of tooth #12 was achieved sequentially first by inverting the lateral bracket upside down and second by giving an individual palatal crown torque in the rectangular wire in relation to tooth #12. Finally proper torque was achieved using a custom-made warren spring (a torquing auxiliary) made with 0.012″ NiTi wire on the rectangular 0.019^″^ × 0.025^″^ SS wire, which moved the root labially and crown palatally [32].

Successful treatment of the complicated case can be achieved by applying appropriate force in the desired direction using conventional biomechanics even without the use of any skeletal anchorage system. Scribante et al. also reported the successful orthodontic repositioning of a transmigrated and lingually positioned mandibular canine using conventional mechanics [33].

## 4. Conclusions

- Multiple impacted teeth with transposition can be successfully managed with controlled tooth movement in three planes of space by applying appropriate force vector using proper biomechanics.
- CBCT is of great value as it provides the precise position and condition of the impacted and transposed teeth. It also guides the direction of traction and avoids interference with the adjacent teeth.
- During alignment of the impacted tooth, the crown of the tooth is readily aligned, but the root remains deviated from the center of the alveolar bone. Therefore, proper torque control is of paramount importance.

## Figures and Tables

**Table 1:** Comparative cephalometric parameters.

Cephalometric parameters	Clinical norms	Pre-treatment values	Post-treatment values
Skeletal parameters			
SNA	82 ± 2°	91°	91°
SNB	80 ± 2°	85°	87°
ANB	2 ± 2°	6°	4°
Wits	0–(−)1 mm	2 mm	1 mm
FMA	25 ± 2°	26°	24°
SN-GoGn	32 ± 2°	29°	28°
Dental parameters			
Max.1-NA (angular)	22 ± 2°	23°	27°
Max. 1-NA (linear)	4 mm	1 mm	2 mm
Max.1-SN	102 ± 2°	113°	116°
Man.1-NB (angular)	25 ± 2°	26°	27°
Man. 1-NB (linear)	4 mm	5 mm	6 mm
LI-A-Pog	2.7 ± 1.7 mm	3 mm	4 mm
IMPA	90 ± 2°	89°	91°
Interincisal angle	134°	126°	124°
Soft tissue parameters			
Nasolabial angle	102 ± 8°	105°	103°
S line to upper lip	0 mm	2 mm	2 mm
S line to lower lip	0 mm	2 mm	1.5 mm
E line to lower lip	−2 mm	1 mm	0.5 mm

## Data Availability

Data supporting this research article are available from the corresponding author or first author on reasonable request.
